# Cholesterol Protects Against Acute Stress-Induced T-Tubule Remodeling in Mouse Ventricular Myocytes

**DOI:** 10.3389/fphys.2018.01516

**Published:** 2018-11-12

**Authors:** Azadeh Nikouee, Keita Uchida, Ian Moench, Anatoli N. Lopatin

**Affiliations:** Department of Molecular and Integrative Physiology, University of Michigan, Ann Arbor, MI, United States

**Keywords:** mouse ventricular myocytes, t-tubule, cholesterol, osmotic stress, potassium currents

## Abstract

Efficient excitation-contraction coupling in ventricular myocytes depends critically on the presence of the t-tubular network. It has been recently demonstrated that cholesterol, a major component of the lipid bilayer, plays an important role in long-term maintenance of the integrity of t-tubular system although mechanistic understanding of underlying processes is essentially lacking. Accordingly, in this study we investigated the contribution of membrane cholesterol to t-tubule remodeling in response to acute hyposmotic stress. Experiments were performed using isolated left ventricular cardiomyocytes from adult mice. Depletion and restoration of membrane cholesterol was achieved by applying methyl-β-cyclodextrin (MβCD) and water soluble cholesterol (WSC), respectively, and t-tubule remodeling in response to acute hyposmotic stress was assessed using fluorescent dextran trapping assay and by measuring t-tubule dependent I_K1_ tail current (I_K1,tail_). The amount of dextran trapped in t-tubules sealed in response to stress was significantly increased when compared to control cells, and reintroduction of cholesterol to cells treated with MβCD restored the amount of trapped dextran to control values. Alternatively, application of WSC to normal cells significantly reduced the amount of trapped dextran further suggesting the protective effect of cholesterol. Importantly, modulation of membrane cholesterol (without osmotic stress) led to significant changes in various parameters of I_K1_, _tail_ strongly suggesting significant but essentially hidden remodeling of t-tubules prior to osmotic stress. Results of this study demonstrate that modulation of the level of membrane cholesterol has significant effects on the susceptibility of cardiac t-tubules to acute hyposmotic stress.

## Introduction

Transverse-axial t-tubular system (TATS) is a complex network of invaginations of the surface membrane necessary for efficient excitation-contraction coupling in cardiac and skeletal muscle cells. In particular, a dense TATS is present in adult ventricular myocytes of likely all mammalian hearts. The TATS becomes significantly remodeled in response to a variety of experimental challenges [e.g., osmotic stress (Kawai et al., 1999; Moench et al., 2013)] and in clinically relevant stress conditions (e.g., heart failure; Guo et al., 2013, for review). It has been well established that a number of t-tubular structural proteins are involved in the maintenance and remodeling of TATS (Kline and Mohler, 2013 for review). However, it is less clear how other membrane components affect TATS structure and remodeling.

T-tubular system membranes are highly enriched in cholesterol (Sumnicht and Sabbadini, 1982) and are stiffer than other biological membranes (Hidalgo, 1985). Cholesterol is an important player in the formation of membrane rafts including caveolae (Simionescu et al., 1983; Rothberg et al., 1990), which can also be found in cardiac t-tubules (Page, 1978). Changes in cellular cholesterol has also been suggested to contribute to electrical remodeling in cardiomyocytes and may have pro- or anti-arrhythmic effects (Coronel, 2017). However, it remains unknown whether in cardiac t-tubules cholesterol exerts its effects through its numerous molecular targets or it can affect their structural stability. Interestingly, patients undergoing statin therapy sometimes experience myalgia associated with skeletal muscle t-tubular dilation and vacuolation (Mohaupt et al., 2009). These skeletal muscle t-tubular defects could be recapitulated *in vitro* in isolated human and mouse muscle fibers by extracting cholesterol using methyl-β-cyclodextrin (MβCD) (Draeger et al., 2006). Recent work has shown that modification of cholesterol content in cardiomyocytes affects cardiac TATS structure (Zhu et al., 2016) although another report has contradicted this initial finding (Gadeberg et al., 2017). One potential underlying reason contributing to this controversy is that these studies, whether using whole animal or isolated cell models, are focused on the analysis of the final result of long term remodeling of TATS and thus may miss the immediate and largely hidden changes in the TATS properties.

Given the important structural role that cholesterol plays in TATS, we hypothesize that one of the likely early consequences of membrane cholesterol modification could be a change in the susceptibility of TATS to various stresses. In this study we show that manipulation of membrane cholesterol leads to significant changes in the susceptibility of TATS to hyposmotic challenge while having no significant changes in the overall appearance of TATS. Furthermore, cholesterol modulation causes significant changes in the electrophysiological properties of cardiomyocytes reflecting diffusional accessibility of TATS that may indicate changes in the underlying t-tubular structure.

## Materials and Methods

### Animals

This study was carried out in accordance with the recommendations provided in the Guide for the Care and Use of Laboratory Animals (8th edition; The National Academic Press, Washington, DC, United States). The protocol was approved by the veterinary staff of the University Committee on Use and Care of Animals at the University of Michigan.

Two- to six-months old male and female C57BL/6 mice were included in this study.

### Solutions (mM)

All solutions were filtered using a 0.22 μm filter and pH adjusted to 7.35 with NaOH. Osmolarity was measured in previous study (Uchida et al., 2016) using a Vapro 5520 osmometer (Wescor, France; mean ± standard deviation; sample size = 3).

Modified Tyrode’s solution (Tyr; 281 ± 4 mOsm/l): 137 NaCl, 5.4 KCl, 0.5 MgCl_2_, 0.3 CaCl_2_, 0.16 NaH_2_PO_4_, 3 NaHCO_3_, 5 HEPES, 10 glucose.

Myocyte storage solution (C solution; 290 ± 3 mOsm/l): 122 NaCl, 5.4 KCl, 4 MgCl_2_, 0.16 NaH_2_PO_4_, 3 NaHCO_3_, 15 HEPES, 10 glucose, 5 mg/mL of bovine serum albumin, 1.38 mg/mL taurine.

Hyposmotic Tyrode’s solution (0.6 Na; 186 ± 3 mOsm/l): prepared as Tyr but with 60% of NaCl.

Hyposmotic (0.7 Na; 211 ± 2 mOsm/l) solution: prepared by mixing 0.6 Na and Tyr solution in a 3:1 ratio.

### Chemicals

HEPES (Calbiochem, United States); KCl, NaHCO_3_, NaH_2_PO_4_ (Mallinckrodt Chemicals, United States); 3 kDa tetramethylrhodamine dextrans in anionic, lysine fixable form (Thermo Fisher Scientific Inc., Waltham, MA, United States). Collagenase (Type 2) (Worthington Biochemical Corp., Lakewood, NJ, United States). Methyl-β-cyclodextrin (MβCD; C4555), Water Soluble Cholesterol (WSC; C4951), Filipin (F9765) and all other chemicals and reagents were purchased from Sigma, St. Louis, MO, United States.

### Isolation of Ventricular Myocytes

Myocytes were isolated from the hearts essentially as described in the study by Moench and Lopatin (2014) and used for experiments within 1–8 h post-isolation.

### Cholesterol Modulation

Methyl-β-cyclodextrin was dissolved in C solution at concentrations of 4, 8, and 12 mg/mL, corresponding to approximate concentrations of 3, 6, and 9 mM. WSC was dissolved in C solution at 5 mg/mL, corresponding to approximately 50 μM cholesterol. All experiments, unless specifically noted, were carried out at room temperature (RT, ≈19–22°C). One of the main reasons for using RT vs. 37°C was to minimize damaging effects of stronger and faster cholesterol depletion at higher temperatures, as well as to reduce general stress on cardiomyocytes.

### Filipin Staining

Quantification of membrane cholesterol was performed using staining with filipin with all procedures carried out at RT. Cardiomyocytes were fixed for 20 min using 2% paraformaldehyde, washed with paraformaldehyde-free solution and incubated with 65 μg/mL filipin for 20 min. After washing out filipin cardiomyocytes were imaged on Nikon TE 20000 microscope using 60× oil immersion objective (NA = 1.4) and CoolSnapEZ camera (Photometrix, Tucson, AZ, United States). Filipin was excited at 340 nm and the emission was collected using 400 nm dichroic mirror and no emission filter in the light path. The data from individual images were corrected for system background, average fluorescence of filipin-free cardiomyocytes (<1–3% of the useful signal) and time-dependent decline in filipin fluorescence due to its washout from the cells (∼30% per hour).

### Electrophysiological Measurements

Ionic currents were recorded in the whole-cell configuration essentially as described in a previous study (Uchida et al., 2016). In brief (Figures 2A,B), accumulation of K^+^ in TATS was induced by applying 400 ms depolarizing step to +50 mV in order to activate voltage-dependent K^+^ currents. Repolarization back to the holding potential of -75 mV leads to appearance of inward-going I_K1_ current, known as I_K1,tail_, originating due to transiently increased concentration of K^+^ in TATS. The density of I_K1_ itself was quantified by measuring the peak of I_K1_ in response to 400 ms voltage step to -120 mV prior to the following voltage ramp (Figure 2A). The ramp data were not used for any analysis and are presented here just for illustration purpose. Initial fast decline of I_K1_ from its peak value (black dot in Figure 2A) is due to depletion of t-tubular K^+^ but not due to change in membrane potential which follows later as a ramp. In order to minimize the variation of various parameters due to the highly variable size of cardiomyocytes the data were normalized to the cell size (cross-sectional, XY, area).

Patched cardiomyocytes were imaged with a MD500 microscope eyepiece camera and the AmScope 3.7 software (AmScope, Irvine, CA, United States). The cross-sectional area was calculated by manually outlining the cell border using *ImageJ*^1^.

### Time Lapse Cell Width Measurements

Control and MβCD treated (4 mg/mL in C solution for 1 h) cardiomyocytes were plated onto a RC-20 perfusion chamber (Warner Instruments, Hamden, CT, United States). Only cells that settled in the center of the perfusion lane were imaged. Images were obtained at 10 s intervals. After a short (∼5 min) period of washout of C solution with Tyr, the perfusion was switched to 0.6 Na solution for 7 min.

The kinetics of solution exchange in the center of the perfusion chamber was measured by recording the change in fluorescence when switching from distilled water to distilled water containing 1:200 diluted 3 kDa dextran. After switching solutions, there is a delay of 3–5 s depending on the location in the bath chamber. The time course of fluorescence change was characterized by a time constant of <500 ms when fit with a single exponential function.

In response to hyposmotic stress, cardiomyocytes behave as nearly perfect osmometers and increase intracellular volume by expanding in width rather than in length (Drewnowska and Baumgarten, 1991). Furthermore, the cell depth to cell width ratio remains constant during hyposmotic swelling (Ogura et al., 2002), indicating that changes in the cell width serve as a good measure of cell volume changes. Experimentally, cardiomyocyte cell width was calculated from time-lapse image stacks using a combination of custom *ImageJ* and *MATLAB* scripts.

### Di-8-ANEPPS Labeling of Cardiomyocytes

Stock solution of di-8-ANEPPS was prepared in DMSO at a concentration of 8.4 mM. The stock di-8-ANEPPS was mixed with 20% pluronic acid in 1:1 ratio and the resulting mixture was diluted (1:600) in C solution to prepare the working solution as previously described (Moench et al., 2013). Right before imaging, cardiomyocytes were incubated in the working solution for 15 min followed by washout with C solution without di-8-ANEPPS.

### Dextran Trapping Assay

Dextran trapping assay was performed essentially as described in earlier publications (Moench et al., 2013; Uchida et al., 2016). In brief, 3 kDa dextran was added to a suspension of isolated ventricular myocytes during the swelling phase in hyposmotic 0.6 Na solution in order to fill t-tubules with this fluorescent marker. Cells were then returned to Tyr solution, still containing dextran, and finally extracellular dextran was washed out using normal Tyr. The cells were further washed and stored in C solution on ice prior to confocal imaging. Control myocytes were treated identically except that they were exposed to Tyr solution instead of 0.6 Na solution.

### Confocal Imaging

Confocal imaging was performed in Microscopy and Image Analysis Laboratory (University of Michigan, Ann Arbor, MI, United States) on an Olympus FV-500 microscope using 60× 1.4 NA oil objective. Images of myocytes were manually outlined and mean intracellular fluorescence of trapped dextran per unit area calculated using *ImageJ*. Further data analysis, e.g., correction for background fluorescence, was performed in Microsoft Excel.

### Skeletonization of TATS

Skeletonization of TATS was performed using approach similar to that described in the paper by Guo and Song (2014). Cardiomyocytes were labeled with di-8-ANEPPS and imaged on confocal microscope using 60× 1.4 NA oil objective and 68 nm pixel size. Image analysis was performed using *ImageJ* and custom macro to automate various steps in the procedure. Images were rotated to bring cardiomyocytes to the same (horizontal) orientation, smoothed using 1 pixel Gaussian Blur. Application of “Auto Threshold” using Huang method followed by “Analyze Particles” to exclude all objects smaller than the size of cardiomyocyte produces the outline of the cell. The outlined cell is then filled with black color and the resulting object uniformly reduced in size using “Erode” function thus creating a mask of cell interior (i.e., cell border excluded). Application of cell interior mask to the original smoothed image followed by “Auto Local Threshold” using Otsu method with radius = 20 provides a binary image of TATS. The images were then skeletonized using Skeletonize (2D/3D) plug-in. Separation of axial ant transverse t-tubules was carried out by application of appropriate morphological filters using MorphoLibJ plug-ins. Specifically, radial and axial t-tubule segments were isolated using 3 pixels long lines (structural element) at 90 and 45 degrees (for radial) and 0 and 135 degrees (for axial), respectively. The use of 45 and 135 degrees lines allows for assigning slanted t-tubular segments to either radial or axial group, and because of left/right symmetry of the cell the effects of application of asymmetrical lines (45 vs. 135 degrees) cancel out. It should be noted that segments smaller than 3 pixels will be lost during the above procedure but this does not affect the ratio of radial vs. axial t-tubules. The density of TATS was calculated as the % of t-tubule pixels relative to the total number of pixels in the cell mask.

### Statistics

The data (mean ± standard error) in each experimental series are from at least two heart preparations. Statistical significance was determined using a one-way ANOVA with Bonferroni correction or two-sample *t*-test assuming equal or unequal variances (whichever is appropriate) and considered significant if *p* < 0.05. With some data one-way ANOVA could not be applied (e.g., due to large disparity in variance) and the data were then analyzed using two-sample *t*-tests. In figures, ^∗^, ^∗∗^, ^∗∗∗^ and #, ##, ### correspond to ANOVA or *t*-test with *p*-values of 0.05, 0.01, and 0.001, respectively.

## Results

### Effects of Membrane Cholesterol Modulation on the Overall Organization of TATS

We first tested the tolerance of cardiomyocytes to various concentrations of MβCD at room temperature (Supplementary Figure 1). There were no easily observable changes in TATS appearance at 3 or 6 mM MβCD but at 9 mM MβCD cells displayed clear deterioration of TATS and increased death rate.

In particular, we also found that the treatment of cardiomyocytes with 1 mM MβCD at 37°C (one of the commonly used conditions) is more damaging than that performed with 3 mM MβCD at room temperature (same exposure time; 1 h). Importantly, with 1 mM MβCD at 37°C only few cardiomyocytes survived the following standard hyposmotic detubulation while cells treated with 1 or 3 mM MβCD at room temperature did not show any overt changes in mortality following detubulation.

Accordingly, the following experiments were performed using 3 mM MβCD to minimize the detrimental consequences of cholesterol depletion in order to help unmask the underlying reasons behind its action.

**FIGURE 1:**
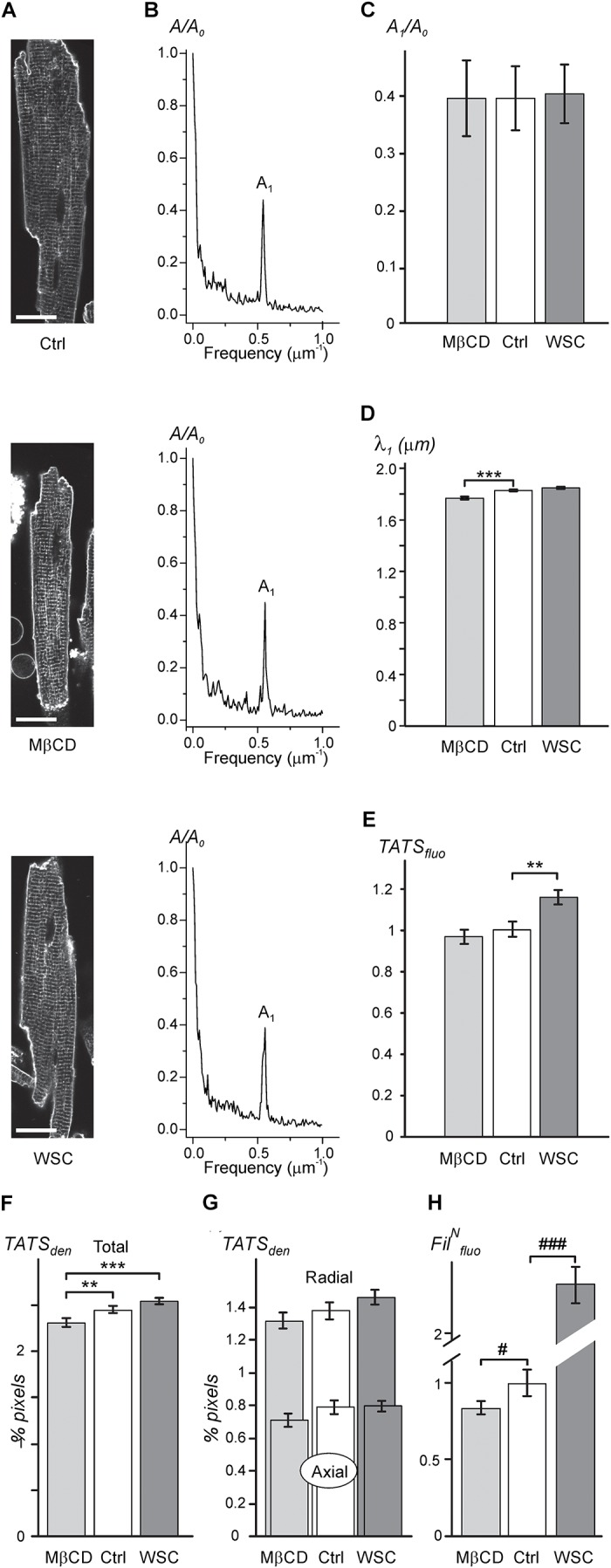
Effects of MβCD and WSC on TATS appearance (**A**; left column). Representative confocal images of control (Ctrl), 3 mM MβCD- and 5 mg/mL WSC-treated ventricular myocytes stained with di-8-ANEPPS. Scale bars: 20 μm. (**B**; middle column). Corresponding amplitude spectra of fluorescence *(normalized to the amplitude of zero order component (*A_0_*). **(C)** Normalized amplitude (*A_1_*) and **(D)** wavelength (λ_1_) of the first harmonic of fluorescence. **(E)** Density of t-tubular fluorescence (*TATS_fluo_*) normalized to that in Ctrl myocytes. **(F,G)** Densities of various components of TATS derived from skeletonization of confocal images of cardiomyocytes labeled with di-8-ANEPPS. The density is calculated as percentage of pixels belonging to TATS skeleton. *n* = 20, 20, and 20 for Ctrl, MβCD- and WSC-treated cells, respectively. **(H)** Effects of MβCD and WSC on the intensity of filipin (cholesterol specific agent) staining. The data are normalized to that obtained in control cardiomyocytes. *n* = 67, 44 and 59 for Ctrl, MβCD- and WSC-treated cells, respectively.)*

As expected, the data in Figures 1A–C show that application of either 3 mM MβCD or WSC (5 mg/mL) has little effect on the overall appearance of TATS. In particular, the regularity of TATS estimated as the amplitude of the first harmonic of fluorescence spectra of membrane bound di-8-ANEPPS dye was not affected by MβCD and WSC (Figures 1B,C). However, quite small (∼3.4%) but statistically significant reduction in the wavelength of the first harmonic corresponding to the sarcomeric length of cardiomyocytes was observed in MβCD-treated cells (Figure 1D). TATS density was estimated in two different ways. First, Figure 1E shows that the intensity of intracellular (t-tubular) fluorescence of di-8-ANEPPS was not affected by MβCD but was increased by ∼16% in cardiomyocytes treated with WSC. Second, skeletonization of the TATS shows no significant effects of the drugs relative to control, although small, but statistically significant difference, can be observed when one would compare the effects of MβCD and WSC (Figure 1F). Additional analysis also shows that the balance between radial and axial t-tubules is not affected as well (Figure 1G). Consistent with relatively low effective concentration of MβCD the fluorescence of filipin, a cholesterol specific agent, was reduced only by ∼12% compared to that in control cardiomyocytes (see section “Discussion” and Supplementary Figure 2 on limitations of filipin-based assay). Importantly, the magnitude of reduction in filipin fluorescence observed with treatment using 3 mM MβCD for 1 h at RT (∼12% reduction; Figure 1H) is significantly smaller than the reduction observed with 1 mM MβCD for 1 h at 37°C (∼50% reduction; Supplementary Figure 2), suggesting that cholesterol extraction treatment in our study is milder than that used in similar studies (e.g., Gadeberg et al., 2017).

Overall, the general appearance of the TATS network remains largely unchanged following cholesterol depletion or supplementation at the indicated concentrations of cholesterol modifying agents.

### Electrophysiological Characterization of the Effects of Membrane Cholesterol Modulation

**FIGURE 2:**
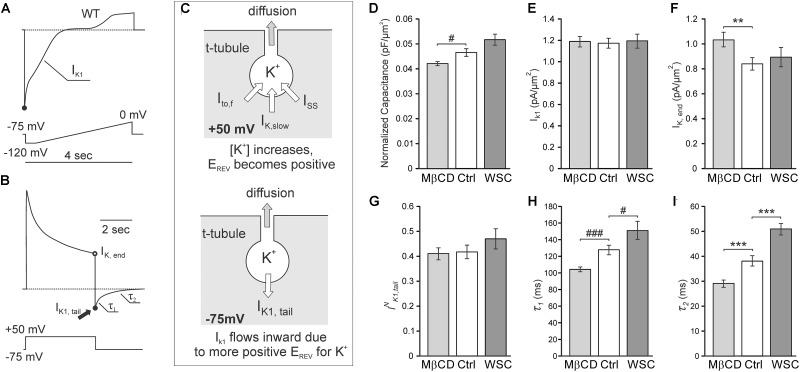
Electrophysiological properties of cholesterol depleted and supplemented cardiomyocytes. **(A)** Cartoon trace of I_K1_ current in response to a slow ramp protocol. **(B)** Cartoon trace of outward K^+^ currents in response to a 400 ms step depolarization to +50 mV and inward I_K1,tail_ in response to a step repolarization back to –75 mV. The amplitude of I_K1,tail_ and its kinetics (characterized by time constants τ_1_ and τ_2_) are measures of diffusional properties of TATS. **(C)** Cartoon diagram depicting the origin of the I_K1,tail_. Top: outward potassium currents cause the accumulation of potassium within the t-tubular lumen during depolarization. Bottom: upon repolarization, the accumulated potassium can either flow into the cell through I_K1_ channels producing the I_K1,tail_ or diffuse out of the t-tubular lumen to the extracellular space. **(D–G)** Quantification of the membrane capacitance normalized to the cell cross-sectional area **(D)**, peak I_K1_ current normalized to the cell cross-sectional area **(E)**, steady-state I_K,end_ **(F)**, and I_K1,tail_ amplitude normalized to the steady-state I_K,end_ current. *n* = 35 control, 40 MβCD, and 16 WSC-treated cells. **(H,I)** Quantification of the slow **(H)** and fast **(I)** time constants of I_K1,tail_ decline. *n* = 37 control, 39 MβCD-, and 16 WSC-treated cells.

Many ionic currents originate from ion channels concentrated in TATS. However, the I_K1,tail_ current (I_K1,tail_) is a unique current that is in large degree dependent on the lumenal K^+^ within TATS (Clark et al., 2001; Cheng et al., 2011), which in turn are dependent on their fine geometrical structure (Uchida and Lopatin, 2018). Figures 2A–C explains the origin of I_K1,tail_ and highlights some important features of this current related to TATS structure. In brief, during a prolonged depolarization, outward potassium currents cause potassium ions to flow into the t-tubular lumen and accumulate due to restricted diffusion to the extracellular space (Figure 2C, top). Upon repolarization, the accumulated potassium depletes by (1) flowing into the cell through I_K1_ channels manifesting as the I_K1,tail_ and by (2) diffusing out of the t-tubule lumen to the extracellular space (Figure 2C, bottom). It has been shown that under conditions similar to that used in our experiments (e.g., holding membrane potential -75 mV) depletion of accumulated potassium by diffusion constitutes a large portion of total efflux and thus I_K1,tail_ can serve as a quantitative measure of diffusional component of TATS (Clark et al., 2001).

We first assessed the effects of cholesterol modulation on membrane capacitance. The averaged values of membrane capacitance (177.1 ± 9.3 pF, 168.2 ± 7.7 pF, and 173.5 ± 9.9 pF) and cell size (cross-sectional area; 3821.4 ± 153.6 μm^2^, 4005.1 ± 164.0 μm^2^, and 3388.5 ± 159.1 μm^2^) were not different (*p* = NS by ANOVA) between Ctrl, MβCD, and WSC groups, respectively. In order to better estimate potential changes in specific membrane capacitance, cell size should be taken into account. As shown in Figure 2D, treatment with MβCD significantly decreases normalized (to cell cross-sectional area) membrane capacitance. Conversely, treatment with WSC leads to an increase in normalized membrane capacitance although with no statistical significance (*p* = 0.06). Since cholesterol modulation significantly affects specific membrane capacitance, membrane currents were normalized to cell cross sectional area. Importantly, we found that I_K1_ is unaffected by cholesterol modulation (Figure 2E). In contrast, the outward potassium current at the end of the depolarizing pulse (I_K,end_) was significantly increased by MβCD treatment compared to that in control cells. Somewhat surprisingly, I_K,end_ in WSC treated cells was essentially unchanged (Figure 2F).

Since the magnitude of potassium accumulation in TATS is dependent on the magnitude of outward potassium current, I_K1,tail_ were normalized to I_K,end_ (Cheng et al., 2011). Consistent with the data in Figure 1, which shows that the overall appearance of TATS is essentially unaffected by cholesterol modulation, no significant differences in the amplitude of the normalized I_K1,tail_ were observed as well (Figure 2G). However, the kinetics of I_K1,tail_ decline, which primarily reflects the rate of potassium diffusion out of the t-tubule lumen, was significantly affected (Figures 2H,I). Specifically, cells treated with MβCD display I_K1,tail_ that decline faster than in control cells as both τ_1_ and τ_2_ of the two-exponential fit were decreased. Conversely, WSC treated cells display a slower decline in I_K1,tail_ with both τ_1_ and τ_2_ significantly increased. This data suggests significant changes in fine (sub-microscopic) geometrical structure of TATS which may occur due to modulation of membrane cholesterol.

### Effects of Modulation of Membrane Cholesterol on the Magnitude of Detubulation

**FIGURE 3:**
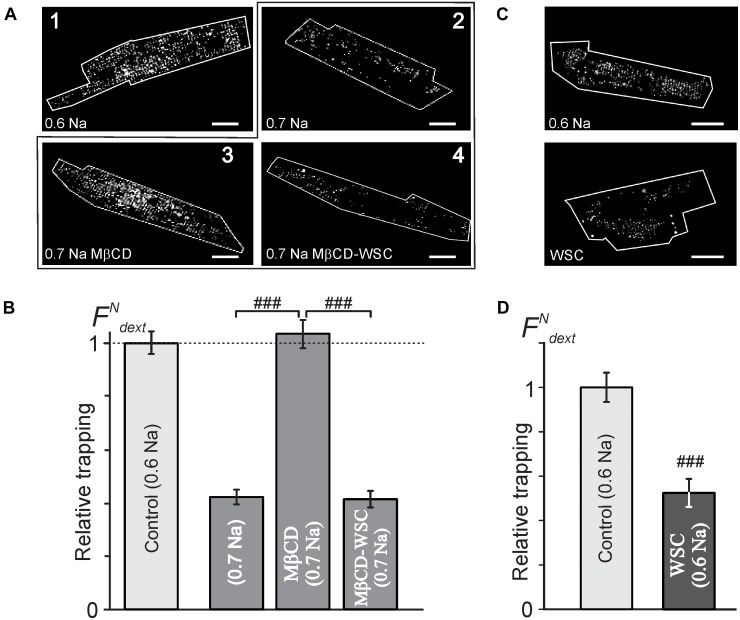
Effects of MβCD and WSC on the amount of dextran trapped in sealed t-tubules. **(A)** Representative confocal images of ventricular myocytes (cell border zone indicated by white outline) highlighting the magnitude of t-tubular sealing in response to hyposmotic shock under various conditions. Detubulation with 0.6 Na solution (1) served as a reference, (2) no treatment, (3) MβCD for 1 h at RT, and (4) 3 mM MβCD for 1 h at RT followed by washout and application of 5 mg/mL WSC for 45 min at RT, all in response to shock using 0.7 Na solution. Scale bar: 20 μm. **(B)** Quantification of the data in panel **(A)**. *n* = 68, 58, 55, and 40 cells, respectively. **(C)** Representative confocal images of control (top) and WSC-treated (5 mg/mL for 45 min) ventricular myocytes detubulated with standard 0.6 Na. **(D)** Quantification of dextran trapping of the data in pane **(C)**. *n* = 35 cells each.

Despite there being no or minor observable effects of cholesterol modifying agents on the overall architecture of TATS (Figure 1) the electrophysiological data above show significant, largely hidden but likely important changes in TATS structure strongly suggesting that there might be other consequences of cholesterol modulation. Specifically, we tested whether susceptibility of TATS to osmotic challenge, known to strongly affect the integrity of TATS (Moench et al., 2013), is affected by MβCD or WSC. In this regard, the magnitude of stress-induced sealing of TATS measured by the amount of trapped extracellular dextran serves as a useful and quantitative measure of TATS stability (Moench et al., 2013). Hyposmotic detubulation with 0.6 Na stress, however, leads to nearly complete detubulation in control cardiomyocytes. In order to maximize the putative observable effect, a milder osmotic stress (0.7 Na) was used to expand the dynamic range of dextran trapping. The data in Figures 3A,B, along with that in Supplementary Figures 3, 4, highlight one of the central findings of this work: depletion of cholesterol by MβCD significantly compromises the resistance of TATS to sealing in response to hyposmotic shock. Importantly, the effect is fully reversible by re-introducing cholesterol with WSC. Significant (>twofold) protective effect of membrane cholesterol is also confirmed in experiments using “full strength” hyposmotic shock with 0.6 Na solution where WSC is applied to normal cardiomyocytes (not previously treated with MβCD; Figures 3C,D).

The data in Supplementary Figure 3 show that cardiomyocytes treated with 1 mM MβCD at 37°C display significantly greater dextran trapping than cardiomyocytes treated with 1 or 3 mM MβCD at room temperature for the same duration. It also follows from the data in Supplementary Figure 3 that treatment with 3 mM MβCD followed by detubulation with 0.7 Na solution places the response in a “dynamic” range allowing for observation of both increases and decreases in dextran trapping.

Notably, the magnitude of dextran trapping in MβCD treated cells inversely correlates with the remaining filipin fluorescence (Supplementary Figure 4), consistent with the notion that membrane cholesterol plays a critical role in TATS susceptibility to osmotic stress.

**FIGURE 4:**
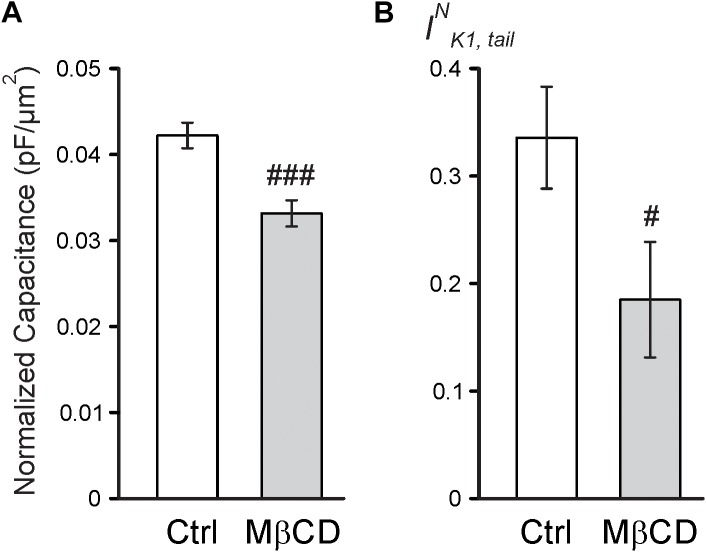
Electrophysiological effects of MβCD after 0.7 Na detubulation. **(A)** Quantification of cell area normalized membrane capacitance of control and MβCD treated cells after detubulation with 0.7 Na. *n* = 13 cells each. **(B)** Quantification of normalized I_K1,tail_ in control and MβCD treated cells after detubulation with 0.7 Na. *n* = 12 and 11 cells, respectively.

Consistent with the above findings, the magnitude of reduction in both normalized membrane capacitance and normalized I_K1,tail_ amplitude (due to sealing of TATS) in response to 0.7 Na hyposmotic stress is more pronounced in MβCD treated cells compared to that in control cells (Figure 4). Overall, the above results suggest that MβCD treated cells are more susceptible to t-tubular remodeling in response to hyposmotic stress.

### Cell Swelling Is Unaffected by Cholesterol Depletion

**FIGURE 5:**
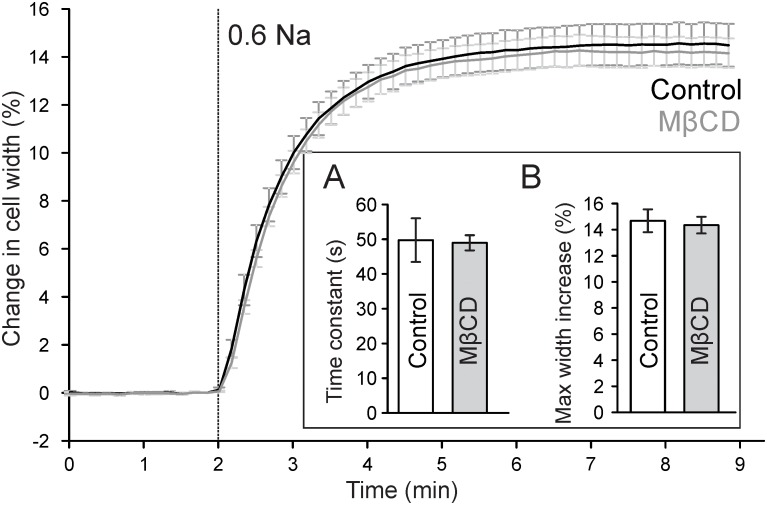
Effect of MβCD treatment on hyposmotic cell swelling. Traces of average changes in relative cell width of control (black) and MβCD treated (gray) cardiomyocytes as a function of time. Vertical line denotes the start of perfusion with hyposmotic 0.6 Na solution. Inset: **(A)** Quantification of the kinetics of cell swelling. A single exponential function was fitted to the time course of cell width during 0.6 Na perfusion and the time constant was averaged for each group (*n* = 7 and 9 for control and MβCD treated cells, respectively). **(B)** Quantification of the maximum change in cell width.

Previous work suggested that cholesterol depletion may accelerate cardiomyocyte swelling in response to osmotic stress (Kozera et al., 2009) and thus may affect the magnitude of changes in TATS following osmotic detubulation. Accordingly, the kinetics of cell swelling in 0.6 Na solution was measured in control and MβCD treated cardiomyocytes. As shown in Figure 5, in both control and MβCD treated cardiomyocytes cell width increases in a nearly identical manner upon perfusion with 0.6 Na solution. The change in cell width can be fitted with a single exponential function and the time constant characterizing the kinetics of cell swelling is not significantly different between those measured in control and MβCD treated cells. Furthermore, there is no significant difference in the magnitude of cell swelling in 0.6 Na solution. These data suggest that cholesterol depletion has no effect on hyposmotic swelling and argues against an osmotic effect underlying the differences in dextran trapping between control and MβCD treated cells.

## Discussion

### Role of Cholesterol in T-Tubular Membranes

The importance of cholesterol in t-tubular membranes is highlighted in recent papers. In particular, cholesterol depletion using MβCD was shown to affect TATS integrity in a concentration and time-dependent manner (Zhu et al., 2016). This effect of MβCD seems controversial as more recent work demonstrated that cholesterol depletion does not appear to disrupt TATS organization (Gadeberg et al., 2017). However, a closer look at the specific experimental conditions in the mentioned studies can reconcile the findings. Specifically, in the study by Gadeberg et al. (2017) threefold lower concentration of MβCD was used compared to the lowest concentration employed in the study by Zhu et al (1 mM vs. 3 mM) (Zhu et al., 2016). The data in our study confirm this view and essentially eliminate that formal discrepancy. Although we performed experiments at RT (vs. 37°C in mentioned papers) a different range of MβCD concentrations was tested.

The data in Figure 1 and Supplementary Figure 1 show that, indeed, at RT and 3 mM MβCD concentration essentially no or only minor changes in the overall structure of TATS can be observed while at 6 mM and higher concentrations the disruptive effect of cholesterol depletion becomes apparent. It should be noted here, that the amount of membrane cholesterol measured using filipin approach is likely overestimated, and thus the effect of MβCD on membrane cholesterol is underestimated (Figure 1H), likely due to membrane permeability of filipin leading to staining intracellular pools of cholesterol (see Supplementary Figure 2 for further detail). Alternatively, the amount of membrane cholesterol may be overestimated in WSC-treated cardiomyocytes likely due to membrane binding of WSC aggregates or micelles.

The two parameters which we found to be affected by modulation of membrane cholesterol were sarcomeric length (Figure 1D; ∼3.4%; MβCD vs. control) and the density of TATS estimated using either di-8-ANEPPS labeling (Figure 1E; ∼19%; control vs. WSC) or skeletonizing algorithm (Figure 1F; ∼10%; MβCD vs. WSC). The change in the sarcomeric length is very small, and the observation became possible in part due to relatively high precision of the measurements employing Fourier transformation. The effect is likely linked to changes in the concentration of resting intracellular Ca^2+^, however, it was impractical to proceed with further investigation of this phenomenon keeping in mind the magnitude of the changes. Measurements of TATS density using di-8-ANEPPS can be significantly confounded by unpredictable effects of cholesterol modulation on the binding of the dye (likely more than due to minor changes in the resting membrane potential of cardiomyocytes), and therefore the data should be interpreted with caution. In contrast, skeletonization approach using local thresholding algorithms is not sensitive to the variation in the intensity of di-8-ANEPPS. Changes in TATS density measured with this approach are very small and could only be reliably detected by comparing two extreme treatments (MβCD vs. WSC). We speculate that the observed effects are not due to true changes in TATS density but are rather apparent (i.e., algorithm dependent) changes due to submicroscopic transformations in the morphology of individual t-tubules. Overall, quantitative data show that the appearance of TATS is essentially unaffected by treatment with MβCD and WSC.

Importantly, however, one has to make a clear distinction between the overall TATS *integrity* and its *stability*. In this regard, one of the central findings of our study is that although the TATS *integrity* appears essentially unaffected following cholesterol modulation with 3 mM MβCD, the *stability* of the TATS in response to stress is greatly compromised. In particular, the data in Figure 3 demonstrate that cholesterol depletion promotes dextran trapping following hyposmotic detubulation, while cholesterol supplementation protects against osmotic detubulation. These data suggest that cholesterol plays a significant role in stabilizing t-tubules against osmotic stress. Importantly, as shown in Figure 5, modulation of membrane cholesterol did not affect the magnitude and kinetics of osmotic swelling suggesting that the observed results cannot be simply explained by changes in mechanical forces caused by osmotic stress. Furthermore, this data may suggest that Zhu et al. observed the combined effects of MβCD treatment and long-term culture, a condition known to cause t-tubule loss (Louch et al., 2004; Zhu et al., 2016; Guo et al., 2018).

### Effects of Cholesterol Modulation on Electrophysiological Properties of Cardiomyocytes

It is commonly expected that cholesterol modulation would have significant effects on membrane capacitance. However, the available data are quite controversial, even in studies using artificial membranes. For example, Ohki (1969) found that capacitance of phosphatidylcholine bilayers increases with addition of cholesterol while in other studies an opposite effect was observed (Naumowicz et al., 2005; Budvytyte et al., 2013), and the effect of cholesterol on membrane capacitance may even show biphasic relationship in lipid monolayers (Pasek et al., 2008). Assuming that increasing membrane cholesterol monotonically decreases membrane capacitance and that cholesterol modulation does not alter the total membrane surface area, one would expect that treatment with MβCD would result in increased membrane capacitance. In contrast, Gadeberg et al. (2017) have recently found that in mouse cardiomyocytes depletion of membrane cholesterol does not alter the membrane capacitance. In even greater contrast, we observe the opposite effect of cholesterol on membrane capacitance (Figure 2). As mentioned earlier, differences in experimental conditions may underlie quantitative differences between the studies. In particular, we used higher concentration of MβCD but lower (RT) temperature which altogether likely led to a stronger modulation of membrane cholesterol. Overall, the data highlight the complexity of cholesterol action on membrane capacitance, which likely involves cholesterol-dependent caveolae that contribute significantly to membrane area (Levin and Page, 1980). In this regard, EM images of cardiomyocytes treated with MβCD display significantly fewer caveolae than control cells (Kozera et al., 2009). However, it remains unknown whether these caveolae are internalized thus decreasing total membrane area or whether the proteins supporting the caveolae structure merely disassemble thus retaining the lipids in the surface membrane. If these caveolae are removed from the total surface membrane, then the membrane capacitance would be expected to decrease, consistent with our findings. Unfortunately, the effect of cholesterol supplementation on the number of caveolae remains unknown and, therefore, it is unclear whether additional cholesterol increases the total surface membrane area as suggested by the membrane capacitance measurements. It should be noted that direct correlation between the intensity of di-8-ANEPPS fluorescence and membrane area cannot be made since di-8-ANEPPS fluorescence is strongly dependent on the presence of membrane cholesterol (Gross et al., 1994).

In contrast to none or relatively small effects of cholesterol modulation on membrane capacitance, there are numerous reports on significant roles of this lipid in the activity of various ion channels (Dart, 2010). In this study we focused on two types of potassium currents highly useful in determining the diffusional properties of TATS: cardiac outward rectifier and inward rectifier potassium currents (Figure 2). We have previously shown that the magnitude of K^+^ accumulation in TATS maximizes at about 400 ms after membrane depolarization and is primarily determined by I_K,end_ which is, in turn, is carried primarily by several members of Kv1 and Kv2 subfamilies of voltage-gated K^+^ channels underlying I_K,slow_ (Nerbonne, 2000). Consistent with previous reports we find that depletion of membrane cholesterol leads to significant increase in late I_K,end_ (Balse et al., 2009), although cholesterol enrichment with WSC did not have any significant effect (Figure 2). Accordingly, because of sensitivity of I_K,end_ to changes in membrane cholesterol, for a meaningful interpretation of the data the magnitude of I_K1,tail_ should be normalized to the magnitude of I_K,end_ (leading to I_NK1,tail_; Figure 2).

As mentioned in Figure 2, I_K1,tail_ dissipates due to two fluxes: movement of potassium into the cell through I_K1_ channels carried by Kir2 subfamily (Anumonwo and Lopatin, 2010) and diffusion of potassium out of t-tubular lumen. Surprisingly, we found that in mouse cardiomyocytes I_K1_ is essentially insensitive to cholesterol modulation (Figure 2E). This is different from the effects observed in endothelial cells endogenously expressing inward-rectifier K^+^ channels (Romanenko et al., 2002) or in cells exogenously expressing Kir2.1 channels (Romanenko et al., 2004) where treatment with MβCD or cholesterol loaded MβCD causes robust changes in the current density. Due to insensitivity of cardiac I_K1_ to membrane cholesterol there was no need to further normalize the I_NK1,tail_ to I_K1_ amplitude.

Consistent with no effects of cholesterol modulation on the overall structure of TATS we found no significant changes in the amplitude of I_NK1,tail_ upon treatment with MβCD or WSC (Figure 2). It should be noted, however, that the interpretation of the I_NK1,tail_ amplitude with regard to TATS structure is not that straightforward, in particular, because it depends on both the density of outward K^+^ current and t-tubular diffusion. In contrast, kinetics of I_K1,tail_ (Figure 2C) depends in a large degree on the diffusional properties of TATS (Clark et al., 2001). In this regard, we recently demonstrated that t-tubular dilations and constrictions, which generally can be viewed as submicroscopic irregularities of t-tubular lumen shape, have a significant impact on the diffusion of molecules within TATS (Uchida and Lopatin, 2018). T-tubule dilations are common features of normal cardiac TATS (Savio-Galimberti et al., 2008; Pinali et al., 2013) and a recent study has reported that t-tubular dilations are associated with regions where Cav3 and RyR2 colocalize (Wong et al., 2013). The loss of these sub-microscopic structures resulting in more uniformly shaped t-tubules would be expected to accelerate the diffusion rate while introduction of more constrictions/dilations will restrict diffusion and slow diffusion rate. The data in Figures 2H,I show that modulation of membrane cholesterol leads to significant changes in diffusional properties of TATS, consistent with the notion that disruption of cholesterol domains [e.g., t-tubular caveolae (Levin and Page, 1980; Burton et al., 2017)] by MβCD results in reduced irregularity of t-tubule diameters and thus faster diffusion of potassium while supplementation with additional cholesterol by WSC does the opposite. An alternative mechanism may involve the effect of cholesterol on t-tubular cBIN1-microfolds which were suggested to contribute significantly to diffusional properties of TATS (Hong et al., 2014). Quantitative analysis of the changes in cBIN1-microfolds, however, would require optical super-resolution or electron microscopy imaging in future projects.

Overall, the results of this study show that in mouse ventricular myocytes modulation of membrane cholesterol leads to significant changes in susceptibility of TATS to acute hyposmotic stress. These findings suggest that long term effects of membrane cholesterol on the integrity of TATS may be explained, at least in part, by its effects of the stability of TATS.

## Author Contributions

AN, KU, and AL designed the research, performed the experiments, analyzed the data, and wrote the manuscript. IM performed the experiments and analyzed the data. All the authors contributed to the final version of the manuscript.

## Conflict of Interest Statement

The authors declare that the research was conducted in the absence of any commercial or financial relationships that could be construed as a potential conflict of interest.

## Footnotes

1 https://imagej.nih.gov
