# Supplementary material for: Cholesterol Protects Against Acute Stress-Induced T-Tubule Remodeling in Mouse Ventricular Myocytes
Source: Front Physiol. 2018 Nov 12;9:1516. doi: 10.3389/fphys.2018.01516 (PMC6240595; doi:10.3389/fphys.2018.01516)
Supplement: Supplementary file 1 [file Data_Sheet_1.pdf]

Supplementary Material

CHOLESTEROL PROTECTS AGAINST ACUTE STRESS-INDUCED T-TUBULE REMODELING IN MOUSE VENTRICULAR MYOCYTES

Azadeh Nikouee[#], Keita Uchida[#], Ian Moench and Anatoli N. Lopatin*

[#] These authors contributed equally to this manuscript.

*** Correspondence:** Corresponding Author: alopatin@umich.edu

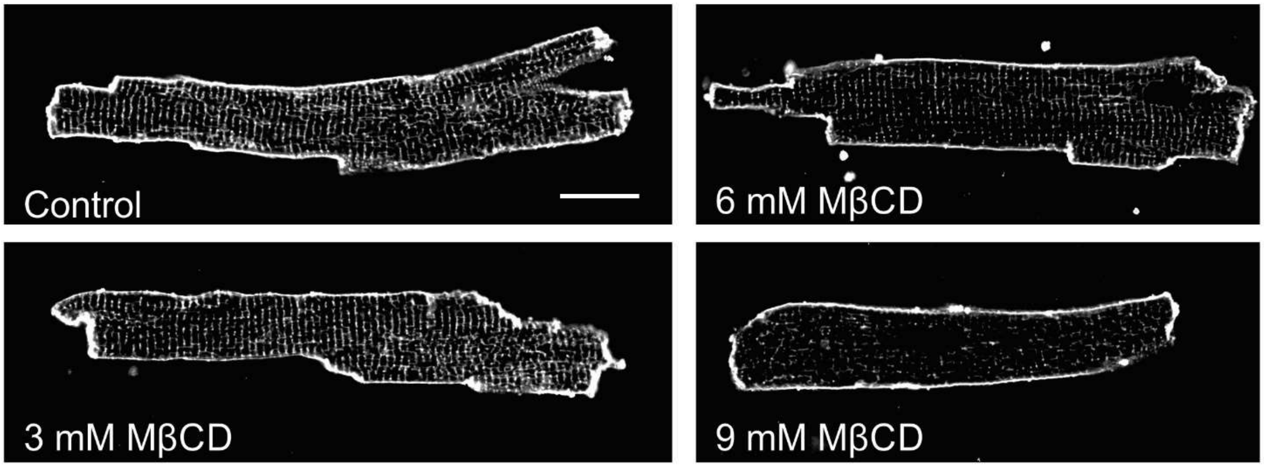

Supplementary Figure 1. Cardiomyocytes were incubated at different concentrations of MβCD at RT for 1 hour followed by membrane labeling with di-8-ANEPPS and confocal imaging. A clear deterioration of TATS was observed a 9 mM MβCD.

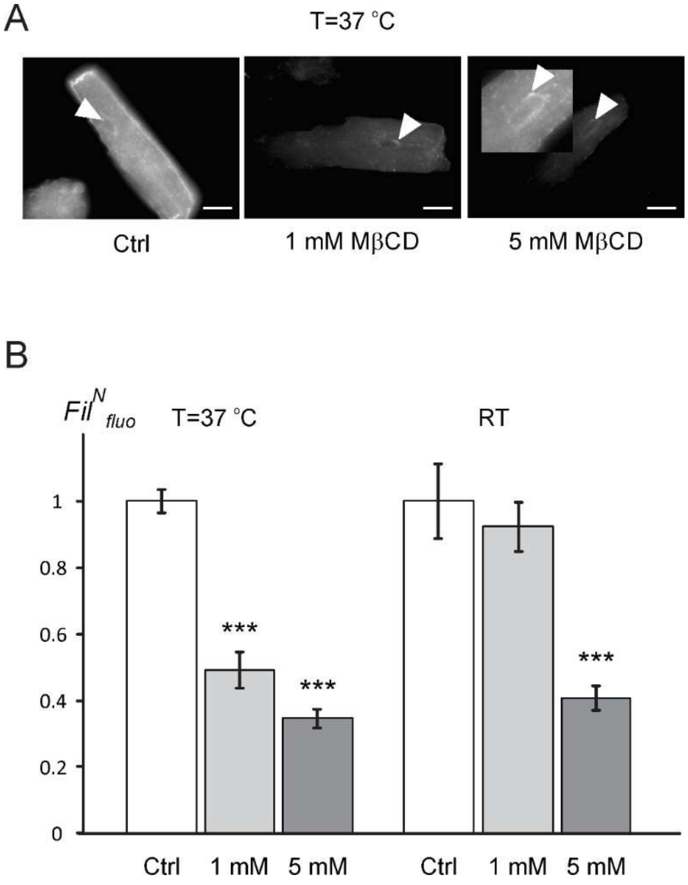

Supplementary Figure 2. Quantification of cellular cholesterol using filipin staining. ***A.*** Representative widefield images of filipin-stained cardiomyocytes. Cells were treated with MβCD at indicated concentrations for 1 hour at 37 ºC. Note there is clear perinuclear fluorescence suggesting that filipin penetrates the membrane and stains intracellular cholesterol, ultimately leading to overestimation of the sarcolemmal component of cholesterol.

B. Quantification of the data. Right panel includes the data from cells incubated at room temperature (RT; ~20-22 ºC). See Methods section in the main text for details. The decrease in the magnitude of filipin fluorescence seems to saturate at high concentrations of MβCD and/or temperature again suggesting significant staining of the intracellular cholesterol (not affected by membrane impermeable MβCD).

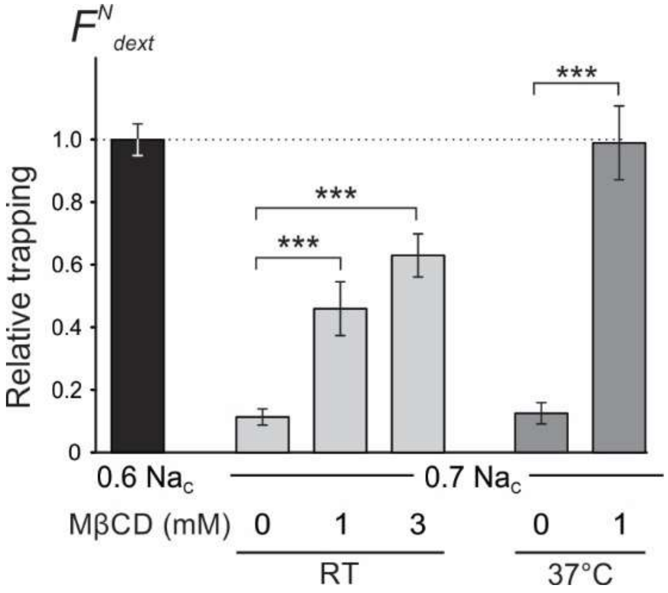

Supplementary Figure 3. Concentration and temperature effects of MβCD on dextran trapping. Cardiomyocytes were incubated for 1 hour at either room temperature (RT; light gray bars) or 37°C (dark gray bars) in C solution containing the indicated concentration of MβCD. Cardiomyocytes treated with 1 mM MβCD at 37°C displayed significant mortality after detubulation with standard hyposmotic Tyrode based solutions (e.g. 0.6 Na). To circumvent this issue, a modified hyposmotic stress protocol was developed using C solution (290 mOsm) as the base solution. Hyposmotic C solutions were prepared containing 60% (0.6 Na_C, 206 mOsm) and 70% (0.7 Na_C, 228 mOsm) of the base [NaCl]. After the 1-hour incubation, cells were detubulated using 0.7 Na_C solution. Control detubulation using 0.6 Na_C solution was performed with cells incubated at RT. All data were normalized to that obtained using 0.6 Na_C solution (black bar). N = 18-20 cells per group.

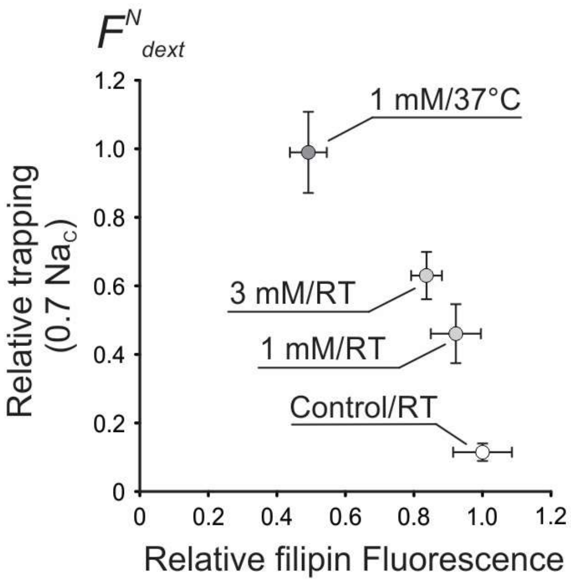

Supplementary Figure 4. Correlation between filipin staining (cholesterol) intensity and magnitude of dextran trapping. The dextran trapping data from Supplementary Figure 3 are plotted against the corresponding filipin fluorescence obtained from Figure 1H and Supplemental Figure 2B.
